# The correlation between serum level of brain natriuretic peptide and amount of left to right shunt

**DOI:** 10.15171/jcvtr.2019.11

**Published:** 2019-02-16

**Authors:** Ahmad Jamei Khosroshahi, Akbar Molaei, Mahmoud Samadi, Elnaz Eskandartash

**Affiliations:** ^1^Cardiovascular Research Center, Tabriz University of Medical Science, Tabriz, Iran; ^2^Pediatric Health Reserch Center, Tabriz University of Medical Science, Tabriz, Iran

**Keywords:** Pro-Brain Natriuretic Peptide, Congenital Heart Disease, Heart Failure, Left to Right Shunt

## Abstract

***Introduction:*** Natriuretic peptides such as brain natriuretic peptide (BNP), atrial natriuretic peptide (ANP) and pro-BNP are secreted in response to atrial and/or ventricular stretch. Left to right shunts such as ventricular septal defect (VSD), atrial septal defect (ASD), and patent ductus arteriosus (PDA), are treated medically or surgically. We aimed to evaluate whether the serum level of pro-BNP would be useful to measure the amount of the shunt.

***Methods:*** In this cross sectional study, 60 infants and children, in whom physical examinations approved heart murmur, and had undergone echocardiography by which VSD, ASD, or PDA had been proven, were included in the study. The relationship between serum BNP levels and severity of shunt (Qp/Qs) based on echocardiographic and hemodynamic evaluations, was studied.

***Results:*** There was a significant relationship between serum level of pro-BNP and the amount of the shunt in the patients with VSD, ASD, and PDA (*P*=0.01). A positive correlation was seen between pro-BNP serum level and Qp/Qs ratio. The mean ± SE serum level of pro-BNP in patients with Qp/Qs ratio of less than 1.5, equal to 1.5-2, and more than 2 was 30.83±2.4, 217.88±44.6, and 217.13±51.8, respectively showing a significant relationship (*P*=0.0001). The cut-off point of pro-BNP demonstrating a Qp/Qs ratio more than 1.5 was measured at the level of 40.36 pg/mL, with a sensitivity and specificity of 92% and 79%, respectively.

***Conclusion:*** Based on our study, the cut-off point of 40.36 pg/mL or more for pro-BNP, showing a Qp/Qs ratio more than 1.5, can be considered as an indication for interventional procedures.

## Introduction

B-type natriuretic peptide is one of the cardiac peptides, which increases in ventricular dysfunction. This substance is produced in the ventricle and released in the form of Pre-Pro brain natriuretic peptide (BNP) and finally becomes degraded enzymatically in response to myocardial stretch to pro-BNP. The serum levels of natriuretic peptides are reliable markers of heart failure and its prognostic value has been shown in many studies.^1,2^

B-type natriuretic peptide is released mainly in the case of ventricular hemodynamic burden and congestive heart failure and is a sensitive and prognostic value of the cardiac performance. BNP is a good marker for the diagnosis of congestive heart failure. Serum level of pro-BNP is increased in systolic and diastolic dysfunctions as it is increased in cases of left to right shunts such as ventricular septal defect (VSD), atrial septal defect (ASD), and patent ductus arteriosus (PDA).^3-5^

Congenital heart diseases with left to right shunt including VSD, ASD, and PDA, constitute about 30% of the cases, which are treated either medically or surgically based on the amount and severity of the shunt and other criteria.^5^ As cardiologists are not available in all parts of the provinces and on the other hand echocardiographic findings are not always sufficient for determining the severity of hemodynamic abnormalities, it seems that by measuring the serum level of B-type natriuretic peptide, the severity of the shunt can be determined and morbidities such as recurrent pulmonary infections, congestive heart failure, failure to thrive (FTT), and Eisenmenger syndrome can be prevented.

Therefore the goal of this study was to evaluate whether serum level of BNP can be used as a marker for determining the amount of the shunt (Qp/Qs), and the severity of the hemodynamic abnormality in congenital heart diseases with left to right shunt such as VSD, ASD, and PDA.^6^

## Materials and Methods

In this cross-sectional study, 60 patients including infants, children, who had gone echocardiography due to heart murmur, recurrent respiratory infection, and FTT, and were diagnosed as having VSD, ASD, and/or PDA were entered into our study.

Exclusion criteria were acute illness (such as sepsis, dehydration, and respiratory disease), heart failure due to cardiomyopathy, myocarditis, and pericarditis, or coronary heart disease.

Using velocity time integral (VTI) measured by Doppler echocardiography at the pulmonary and aortic valves for VSD and ASD, and mitral and tricuspid valves for PDA, the Qp/Qs ratio was calculated using the following formula:

$$\begin{array}{l} Qp=RVOT\;VTI\times \pi \times (\frac{RVOT^{2}}{2})\\ Qs=LVOT\;VTI\times \pi \times (\frac{LVOT^{2}}{2})\\ Qp/Qs\;Ratio=Qp/Qs \end{array}$$

According to Qp/Qs ratio, the severity of the disease were divided into three categories; mild (Qp/Qs <1.5), moderate (1.5<Qp/Qs<2), and severe (Qp/Qs>2)

Finally the pro-BNP level was measured using florescence immunoassay triage kits and compared with the amount of left to right shunt (Qp/Qs), and the correlation was evaluated.

### Statistical Analysis

The data were evaluated using descriptive statistical methods (mean ±SE), incidence, percent, mean differential test in independent groups for quantitative variables and mean chi-square test for qualitative variables. Also we used “*Box and Whisker plots”* for demonstrating the distribution of the data in the subgroups. The statistical analyses were done using SPSS software version 17. *P* value <0.05 was considered as statistically significant.

## Results

Of the 60 patients evaluated, 25 (41.66%) were male and 35 (58.33%) were female. The mean age of the patients was 4.10±0.53 years (max=9, min=0.5). The mean age of the male patients was 3.86±0.23 (max=9, min=0.5) years and that of female patients was 3.2±0.79 (max=9, min=0.53) years. There was significant difference between male and female patients regarding the age. Of the total 60 patients, 20 had ASD (33.3%), 20 had VSD (33.3%), and 20 had PDA (33.3%, Table 1).

**Table 1:** The demographic findings of the patients with left to right shunt

	**The type of left to right shunt**			***P***
	**ASD**	**VSD**	**PDA**	
Patients	20 (33.3%)	20. (33.3%)	20 (33.3%)	-
Age (y)	6.2±0.33	4.2±0.82	3.1±0.16	0.36
Sex				0.87
Male	8 (40%)	7 (40%)	10 (50%)	
Female	12 (60%)	13 (60%)	10 (50%)	
Weight (kg)	18.1±1.11	14.8±3.16	10.9±1.51	0.07

In our study, the mean serum levels of pro-BNP were 30.83±2.4, 161.56±29.71, and 329.02±51.25 for those patients with Qp/Qs <1.5, 1.5<Qp/Qs<2, and QP/QS>2, respectively. There was a significant correlation between the Qp/Qs ratio and BNP serum level (*P*=0.0001) (Table 2).

**Table 2:** Relationship between serum pro- BNP and the QP/QS

	**QP/QS**			***P***_a_	***P***_b_	***P***_c_	**Pro BNP**
	**<1.5**	**1.5-2**	**≥2**				
Patient number (%)	18 (30)	15 (25)	27 (45)	<0.0001*	<0.0001*	<0.0001*	
Shunt							
Mild	18(30)						30.83±2.4
Moderate		15(25)					161.56±29.71
Severe			27(45)				329.02 ±51.25

*P*_a_ *: P* value for QP/QS <1.5.; *P*_b_*: P* value for QP/QS 1.5-2.; *P*_c_*: P* value for QP/QS ≥2.

Also as demonstrated in Table 3, the quantities of pro-BNP had a statistically meaningful correlation with the underlying cause of left to right shunt, in a way that the least amount was detected in PDA, followed by VSD, and ASD.

**Table 3:** Relationship between serum pro- BNP and the QP/QS

	**QP/QS**			***P***_a_	***P***_b_	***P***_c_	**Pro BNP**
	**<1.5**	**1.5-2**	**≥2**				
Patient number (%)	18 (30)	15 (25)	27 (45)	<0.0001*	<0.0001*	<0.0001*	
Shunt							
Mild	18(30)						30.83±2.4
Moderate		15(25)					161.56±29.71
Severe			27(45)				329.02 ±51.25

*P*_a_ *: P* value for QP/QS <1.5.; *P*_b_*: P* value for QP/QS 1.5-2.; *P*_c_*: P* value for QP/QS ≥2.

In our study the serum level of pro-BNP had a significant relationship with the left to right shunt in patients with ASD, VSD, and PDA (*P *= 0.01) (Table 3).

## Discussion

Congenital heart diseases exist in the fetal period but usually have no clinical manifestations. Mostly, they present themselves after delivery in the neonatal period, infancy, childhood, or even later.^4,5^

In the study by Ozhan and colleagues in 2007,^6^ the BNP serum levels were compared with the Qp/Qs ratios taken echocardiographically, and it was shown that in patients with a Qp/Qs ratio <1.5, the mean BNP was 17±14, and in those with a Qp/Qs ratio>1.5, the mean BNP was 56±45, which in turn indicated a significant correlation between the BNP level and the Qp/Qs ratio. Therefore based on this study, serum BNP level can be used as a screening tool for the evaluation of VSD and ASD.^6-9^

In the study done by Law et al,^10^ 33 patients with left ventricular (LV) failure were evaluated. Of them, 22 underwent angiography and 11 patients developed heart failure. The mean serum BNP levels were 754±1.086, 251±169, and 11±5 pg/mL for those with aortopulmonary shunt, cavopulmonary shunt, and control groups, respectively, which was statistically significant (*P*=0.004). Also in this study the mean left end diastolic pressure (8 mm Hg), mean pulmonary arterial pressure (14.5 mmHg) and mean right atrial pressure (6.5 mm Hg) had all significant relationship with serum BNP level.^10-13^

Therefore it seems that the BNP level is a reliable marker for predicting the amount of shunt and hemodynamic impairment and consequently, a cut-off point for serum pro-BNP level is essential.

In a statistical analysis, a significant correlation was seen between pro-BNP level and the underlying cause of left to right shunt (*P*=0.01). These findings show that serum pro-BNP level may indicate the kind of the shunt.

In the study done by Jansle and colleagues, the relationship between BNP level and the hemodynamic findings after catheterization of the patients with VSD and ASD, was evaluated. of 59 patients, 80% had a Qp/Qs> 1.5 and 20% had pulmonary hypertension (14). This result was compatible with ours in which 80% had a Qp/Qs >1.5 and in 20% of the patients it was less than 1.5. In our study, both pro-BNP (*P*=0.166). They determined a cut-off point of 15 pg/mL with a specificity and sensitivity of 100% and 28%, respectively. Therefore they concluded that a BNP>15 pg/mL was more helpful for those patients who needed interventions.^14,15^

In our study the mean level of pro-BNP was 194.9±21.2, and there was a positive significant correlation between pro-BNP levels as the amount of systolic pulmonary artery pressure with the degree of shunt measured by echocardiography (*P *= 0.06).

In our study, the cut-off point for pro-BNP level was determined at 40.36 pg/mL in patients with a Qp/Qs>1.5, with the specificity and sensitivity of 88.3% and 100%. Therefore pro-BNP≥40.36 pg/mL is helpful for determining patients who need interventions.

## Conclusion

As was observed in our study, mean pro-BNP level was correlated with the amount of left to right shunt (Qp/Qs ratio) in children with ASD, VSD, and PDA in a decreased degree of relationship. The serum level of B type natriuretic peptide had a significant correlation with the amount of left to right shunt (Qp/Qs ratio) in patients with ASD, VSD, and PDA (*P *= 0.0001).

The mean pro-BNP was related to Qp/Qs<1.5 or Qp/Qs>1.5 (*P *= 0.0001) and the amount of left to right shunt (*P *= 0.02) in patients with VSD, ASD, and PDA. Therefore pro-BNP can be used as a screening tool for evaluating hemodynamic load in patients with VSD, ASD, and PDA.

Based on our study pro-BNP serum level ≥40.36 mg/dL with the specificity and sensitivity of 100% and 88.3%, respectively, is helpful for detecting the amount of Qp/Qs ratio and the need for more interventions.

## Competing interests

All authors declare no competing financial interests exist.

## Acknowledgments

The authors would like to thank Pediatric Health Research Center, Tabriz University of Medical Sciences, Tabriz, Iran. We would like to thank all patients participating in the study.
